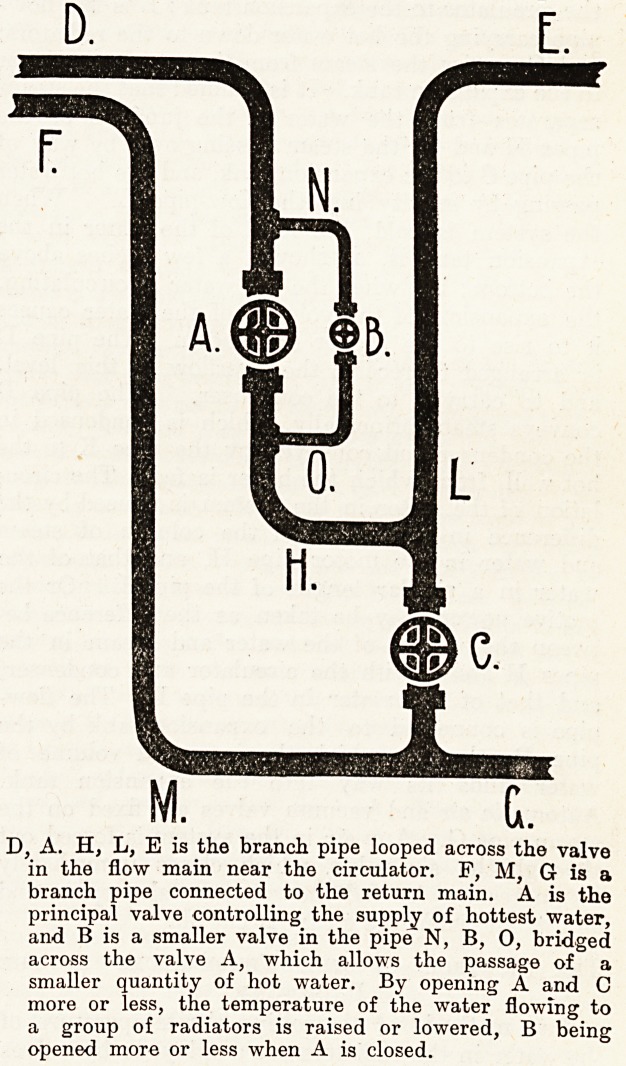# The Western Infirmary, Glasgow

**Published:** 1915-09-11

**Authors:** 


### THE HEATING OF HOSPITALS.

**[NOTE.—In a series of articles the writer proposes to describe the different systems of heating that are employed in hospitals. He will be pleased to answer any questions through "The Hospital," bearing upon the subject of heating or ventilation. Each system will be illustrated and described as exemplified at some hospital where it has been applied. In the first article the method by the use of low-pressure steam employed at Leicester Infirmary was dealt with.]**

### II. The Western Infirmary, Glasgow.

### THE "RECK" SYSTEM OF HEATING BY HOT-WATER RADIATORS.

In this system, the invention of Captain Reck, of Copenhagen, hot water is employed, flowing through the radiators and the connecting pipes at a high velocity, obtained by the aid of a column of hot water and steam, produced by what the inventor calls a "circulator."

The system differs from the ordinary thermo-syphon system in several important particulars. There are the usual flow and return pipes with the

Diagram of the "Reck" System of Hot-water Heating as Fixed at the Western Infirmary, Glasgow.

A, Expansion tank; B, Expansion pipe; C, Surplus steam pipe; D, Overflow pipe; E, Circulator; F, Main return pipe; G, Steam supply pipe; H, Motor pipe; J, Condenser; K, Condenser pipe; L, Main flow-pipe.

radiators bridged between them, and the usual expansion tank; but the flow-pipe is the one in which the water is descending, while it ascends in the return pipe instead of the reverse, as is customary. Also, the expansion tank, in addition to providing for the increased volume of the water in the system when it is heated, has the important office of taking care of the surplus steam and handing it on to the condenser. The "circulator" takes the place of the boiler with small establishments, and the calorifier with large establishments, but the heat is applied by injecting steam directly into the circulating water, instead of by causing steam or hot gases to pass over the outsides of pipes in which the water is circulating. The condenser is designed to prevent the waste of heat in the steam that is not absorbed by the water.

#### The Circulator.

The circulator is a cylindrical vessel, through which the return water passes, just as it passes

D, A. H, L, E is the branch pipe looped across the valve in the flow main near the circulator. F, M, G is a branch pipe connected to the return main. A is the principal valve controlling the supply of hottest water, and B is a smaller valve in the pipe N, B, O, bridged across the valve A, which allows the passage of a smaller quantity of hot water. By opening A and C more or less, the temperature of the water flowing to a group of radiators is raised or lowered, B being opened more or less when A is closed.

through the calorifier or the hot-water boiler. Steam is forced into it at pressures ranging from 2½ lb. per square inch up to 7½ lb. The steam is broken up by a nozzle and by perforated plates, so that it thoroughly mixes with the water. The addition of the steam to the water causes the latter to boil and to ascend, just as the hot gases in a boiler chimney do. The circulator can be placed at any part of the system, but in practice it is fixed a few feet below the expansion tank, which, as usual, is in the upper part of the building. The expansion tank must be above the highest radiator, and the steam must be above the circulator; so in practice a pipe carrying steam at reduced pressure is taken up above the expansion tank and a pipe dropped down from it to the circulator. The condenser is also placed in the return pipe, the water which has done its work in the radiators being led through it before it enters the circulator. Fig. 1 is a diagram of the " Reck " system as it is arranged at the Western Infirmary. The cold water from the radiators passes up the pipe F to the condenser J, and thence to the circulator E; G is the pipe bringing the steam to the circulator, and H is the "motor pipe," as the inventor calls the pipe carrying the hot water and steam from the circulator to the expansion tank; L is the flow-pipe carrying the hot water down to the radiators, and C carries the steam from the motor pipe over to the expansion tank. It is claimed that the steam separates from the water at the junction of the pipes H and L, the steam passing over by way of the pipe C to the expansion tank, and the hot water passing by gravity into the flow-pipe L. When the system is cold, the level of the water in the expansion tank is, as shown, a few inches above the bottom; but when the hot water is circulating, the expansion of the volume of the water causes it to rise to the higher line shown. The pipe D is arranged to receive the overflow at this level, and to carry it to the condenser. The pipe D conveys steam principally, which is condensed in the condenser and conveyed by the pipe K to the hot well, from which the boiler is fed. The circulation of the water in the system is caused by the difference in the weight of the column of steam and water in the motor pipe H, and that of the water in a similar length of the pipe L. Or the motive power may be taken as the difference between the weight of the water and steam in the pipes H and F with the circulator and condenser, and that of the water in the pipe L. The flow-pipe is connected to the expansion tank by the pipe B, through which the increased volume of water finds its way into the expansion tank. Automatic air and vacuum valves are fixed on the steam pipe G. Any air in the system is forced out through the air valve, which closes immediately steam reaches it, and the vacuum valve opens and admits air when the steam is cut off.

The Control of the Temperature in the Radiators.

Two methods of controlling the temperature of the water in the radiators are employed: by valves at each radiator, enabling the rate of flow of water through the radiator to be increased or decreased at will, and by what the inventor has termed " mixers."

The temperature of the water in the pipes varies in different portions of the pipes; just beyond the circulator it is highest, just before it enters the condenser it is lowest. This is taken advantage of in the " mixers " to regulate the temperature in any group of radiators.

A loop of pipe is connected to two parts of the flow-pipe near the circulator, between which a valve is inserted, and the loop is led to a convenient part of the building. Connections are also made with the return pipe, so that by means of the valves, as shown in fig 2, water at higher or lower temperatures can be directed to any group of radiators. By decreasing the flow from the pipe carrying hot water and allowing a certain quantity to flow from the cold pipe, the temperature in the group of radiators is lowered, and by increasing the flow of hot water it is raised, so that any desired temperature, within the range of the system, can be obtained.

The apparatus was fitted up by Messrs. James Boyd and Sons, of Paisley, under the direction of Colonel Mackintosh. The writer understands that Messrs. Boyd are the licensees for the system in this country; they have kindly supplied the diagrams.

## Figures and Tables

**Figure f1:**
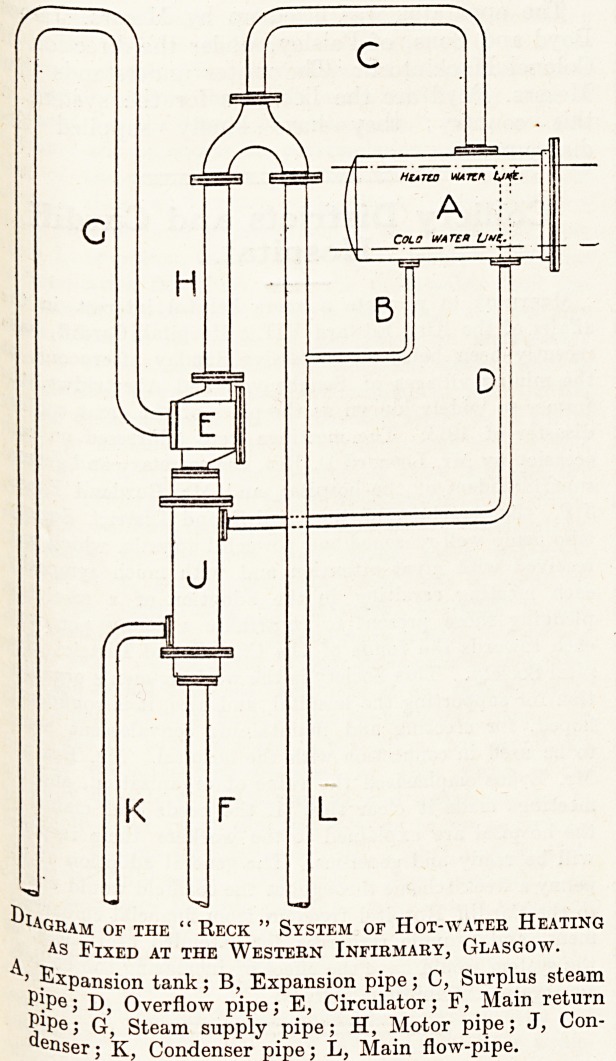


**Figure f2:**